# Ethnicity and paediatric healthcare utilisation: Improving the quality of quantitative research: Claire Zhang

**DOI:** 10.1093/eurpub/ckac129.729

**Published:** 2022-10-25

**Authors:** CX Zhang, MA Quigley, C Bankhead, T Bentley, C Otasowie, C Carson

**Affiliations:** 1 National Perinatal Epidemiology Unit, University of Oxford, Oxford, UK; 2 Department of Primary Care Health Sciences, University of Oxford, Oxford, UK; 3 Medical Sciences Division, University of Oxford, Oxford, UK

## Abstract

**Background:**

The COVID-19 pandemic highlighted the stark health inequities affecting minority ethnic populations in Europe. However, research on ethnic inequities and healthcare utilisation in children has seldom entered the policy discourse. A scoping review was conducted in the UK, summarising and appraising the quantitative evidence on ethnic differences (unequal) and inequities (unequal and unfair or disproportionate to healthcare needs) in paediatric healthcare utilisation.

**Methods:**

Embase, Medline and grey literature sources were searched for studies published 2001-2021. Studies that found differences and inequities were mapped by ethnic group and healthcare utilisation outcome. They were appraised using the National Institute for Health and Care Excellence appraisal checklists. The distribution of studies was described across various methodological parameters.

**Results:**

Of the 61 included studies, most found evidence of ethnic variations in healthcare utilisation (n = 54, 89%). Less than half attempted to distinguish between ethnic differences and inequities (n = 27, 44%). Studies were concentrated on primary and preventive care and hospitalisation, with minimal evidence on emergency and outpatient care. The quality of studies was often limited by a lack of theory underpinning analytical decisions, resulting in conflation of difference and inequity, and heterogeneity in ethnic classification. The majority of studies examined children's ethnicity but overlooked parent/caregiver ethnicity, and also didn't investigate patterns across age, year or location.

**Conclusions:**

To improve the validity, generalisability and comparability of research on ethnicity and paediatric healthcare utilisation, findings from this scoping review were used to develop recommendations for future research. These lessons could be applied more broadly across the European context to improve evidence generation and evidence-based policy-making to reduce inequities in healthcare.

**Key messages:**

• Quantitative studies of ethnicity and paediatric healthcare utilisation in the UK lack the use of sound theoretical frameworks, and often do not distinguish between ethnic differences and inequities.

• The quality of future studies can be improved with greater attention to how ethnicity is classified and analysed, alongside specific considerations for examining healthcare utilisation in children.

